# Supplementary material for: HAPP: High-accuracy pipeline for processing deep metabarcoding data
Source: PLoS Comput Biol. 2025 Nov 7;21(11):e1013558. doi: 10.1371/journal.pcbi.1013558 (PMC12622834; doi:10.1371/journal.pcbi.1013558)
Supplement: S1 Table — Species and BOLD BIN numbers are provided both based on individual ASV annotations and based on consensus (‘cons.’) annotations of OTUs. OTU clustering is applied only after chimera removal in HAPP, which explains the NA values. ‘After_cleaning’ refers to the last (optional) cleaning step where OTUs corresponding to spike-in controls and OTUs occurring in more than a specified fraction of negative control samples are removed. (PDF) [file pcbi.1013558.s006.pdf]

	Reads	ASVs	OTUs	Species	BOLD BINS	Species cons.	BOLD BINS cons.
**Sweden**							
Unprocessed	4605063495	821559	NA	11253	23745	NA	NA
After_chimera	4604593870	699265	119109	11238	23669	10565	20238
After_NEEAT	4445347636	516775	34047	11097	23100	10393	19575
After_cleaning	4444962027	516102	33995	11094	23097	10390	19572
**Madagascar**							
Unprocessed	1494709009	701769	NA	2459	9193	NA	NA
After_chimera	1494552305	688534	231136	2455	9182	2384	8292
After_NEEAT	851470856	356262	77639	1755	7730	1693	6874
After_cleaning	839914457	354870	77604	1744	7715	1683	6860
