# Supplementary material for: HAPP: High-accuracy pipeline for processing deep metabarcoding data
Source: PLoS Comput Biol. 2025 Nov 7;21(11):e1013558. doi: 10.1371/journal.pcbi.1013558 (PMC12622834; doi:10.1371/journal.pcbi.1013558)
Supplement: Attachment — Submitted filename: HAPP_responses_to_reviewers_v2.pdf [file pcbi.1013558.s009.pdf]

Dear Dr. Chen and Dr. Bollenbach,

Thank you for forwarding the comments from the reviewers and for giving us a chance to submit a revised version of our manuscript to PLOS Computational Biology.

We have addressed all the reviewer comments below and in the revised manuscript (changes are marked in yellow in the 'Revised Article with Changes Highlighted' document).

As agreed in the email correspondence, we have changed article type from ‘Software’ to ‘Methods’, and have made sure that the manuscript adheres to the organization of a Methods article.

Thank you for your consideration of this work.

Sincerely yours,

Anders Andersson and Fredrik Ronquist

Reviewer #1

The study by Sundh et al. describes a pipeline for processing metabarcoding data, primarily focused on insect communities using coding sequences (specifically CO1). The pipeline, HAPP, integrates previously published bioinformatic tools and introduces a new algorithm, NEEAT, to improve denoising. The benchmarking results are informative to implement the best tools and parameters as well. However, I have several questions regarding the pipeline:

Reply: We appreciate the insightful comments provided by the reviewer and address each of them below.

- What is the purpose of using DADA2 before clustering? Have the authors tested the pipeline without this step to evaluate whether DADA2 is truly necessary in this context?

Reply: This is a good point. We now address it in a new paragraph in the Discussion: "Since HAPP clusters the data into OTUs, one might argue that first denoising the raw sequence data into ASVs is not necessary. However, there are several merits of inferring ASVs. ASVs make it faster and easier to reanalyse data at a later state - for example reclustering into OTUs, updating taxonomic annotations, and merging ASV tables from different studies - compared to starting with the raw data. Moreover, ASVs facilitate analyses of intra-specific diversity."

- Figure 5 is confusing. The pipeline generates ASVs, optionally removes chimeras, assigns taxonomy, then clusters into OTUs and removes noise using NEEAT. Assigning taxonomy before clustering and denoising seems counterintuitive and potentially time-consuming. Could the authors clarify their rationale for this order of operations?

Reply: The pipeline does not generate ASVs, ASVs with their counts in samples are the input to the pipeline, as the legend of Figure 5 indicates. Doing taxonomic annotation on the individual ASVs is indeed more time consuming than only once per OTU. However, we use the taxonomic annotation to split up the ASVs into taxonomic groups in the clustering and noise filtering steps, which greatly reduces the computation time and memory usage of these steps. In addition, the taxonomic assignment steps are also parallelised by splitting the input sequences into chunks of configurable size (500 sequences by default).

- I am also puzzled by the choice of SINTAX over VSEARCH, especially given that the authors mention VSEARCH performed better. In most community ecology studies, we lack complete knowledge of all species present in a community. Therefore, it would make more sense to prioritize tools that perform best when genus or species information is missing. Could the authors clarify this choice? Additionally, what is the proportion of incorrectly assigned ASVs (at the species, genus, and family levels) that were nonetheless assigned to the closest taxon in terms of phylogeny? In other words, how many ASVs were incorrectly assigned when the correct species was absent, but were assigned to the most closely related species available in the database?

Reply: As we write in the Results and show in Figure 2, SINTAX clearly outperformed VSEARCH when the same species was present in the reference database. VSEARCH had a higher number of correctly assigned at the genus level when the species was missing, and at the family level when the genus was missing, but simultaneously higher numbers of incorrect assignments than SINTAX. Since SINTAX worked well at the species level while reporting low false identifications at the higher levels, we chose that method, in combination with the phylogenetic method for order level. But note that HAPP allows using any of the methods included in the benchmarking.

- While the pipeline is interesting, it appears to have limited applicability. Is there a version of the pipeline that can be used with SSU rDNA, which is the most commonly used marker? Can it be used on another set of taxa for example eDNA from marine samples?

Reply: In principle HAPP can be used on any marker gene. But it is more relevant for markers displaying intra-specific diversity, such as CO1 and ITS. For SSU rDNA (16S and 18S), often the ASV level is required to distinguish species (and sometimes even this level fails to resolve species). Furthermore, the main benefit of the NEEAT algorithm is to remove NUMTS, and these do not exist for SSU rDNA. Although SSU rDNA sequencing has been the most widely used application of metabarcoding, the method is extensively used for insects and other animals using the CO1 gene.

I have also a few questions about NEEAT:
- What happens if only a limited amount of data is available for reference, especially for the Evolutionary signature part?

- How do you make the difference between error and population variation?

Reply: Both of these questions are now addressed in a new paragraph in the Discussion: "NEEAT is designed for deep metabarcoding data and may be less effective for smaller datasets. For instance, NEEAT separates NUMTs and sequencing errors from biological sequence variants by looking for the distribution of read ratios across samples. Authentic sequence variants are likely to be associated with significant spatial or temporal variation in read ratios, unlike NUMTs and sequencing errors. The more samples, the easier to separate these patterns. Similarly, a more complete sample of the fauna or flora facilitates the identification of NUMTs and sequencing errors through the deviations in pairwise evolutionary signatures used by NEEAT."

- Why DADA2 followed by clustering do not eliminate the noise? What do you mean by recurring sequencing error?

Reply: Our analyses clearly illustrate that DADA2 with default settings followed by clustering does not completely eliminate noise from chimeras, sequencing errors or NUMTs in our benchmark dataset. The word "recurring" in this context refers to the fact that the errors that are filtered out by NEEAT may be likely to occur in more than one sample. However, errors may also be unique to one sample, and the distinction is really not important here. To avoid confusion, we removed "recurring".

Minor comments:
I recommend that the authors cite the original publications describing the tools listed in Table 2, along with their respective versions, rather than only referencing the version of QIIME2. For example, the authors should cite Rognes et al. (2016) for VSEARCH instead of citing the QIIME2 publication, and should use the latest version of the tool (v2.30). I was unable to determine which version of VSEARCH is implemented within QIIME2.

Reply: Rognes et al. (2016) is now cited and the VSEARCH version is specified in the 'qiime_vsearch' line of Table 2.

Some references are needed for example L 90-93.

Reply: We have now added references in this section and have tried to improve the clarity by rephrasing the text on lines 88-90.

Reviewer #2

The manuscript by Sundh and collaborators presents a pipeline (HAPP) for processing denoised amplicons from deep (>2M reads/sample) metabarcoding, including a new process, NEEAT, that eliminates spurious sequences.

The authors test their work with two datasets of insect samples metabarcoding, from Sweden and Magadascar, using a fragment of the COI gene. The pipeline is available on github.

The manuscript explains the particularities of deep metabarcoding, although it does not mention the effect of the binned Qscores offered by the Illumina platforms used for deep metabarcoding. The starting point of the HAPP is the ASV table from dada2, but the estimation of the error rates in which dada2 relies for its denoising is not informative when only 3 or 4 Qscores are given. But using DADA2 is, I think, irrelevant for this pipeline. The default setup for secondary clustering in HAPP (using swarm) is d = 12. That implies that sequences with up to 12 unseen haplotypes linking them will end up in the same OTU. It is very, very unlikely that DADA2 is doing anything in this regard: raw sequences will have way fewer errors corrected by DADA2 (this info can be extracted from dada2). In other words, I think doing dada2 + swarm with a high d offers the same output as swarm with a high d from the derep data. Particularly if the error object is not very useful.

For the purposes of my review, I will ask the authors to address the Qbinned scores and their effect in ASV estimation. I think it makes a great point in favor of using HAPP, given that the ASV estimation on its own might be misleading.

Reply: We want to clarify that the HAPP pipeline does not generate ASVs. The pipeline can in principle use any sequences in fasta format as input. Above we explain why using ASVs rather than clustering on the reads directly is motivated. While there is an ongoing discussion about binned quality scores on the DADA2 GitHub issue tracker (see eg: https://github.com/benjjneb/dada2/issues/1307 and https://github.com/benjjneb/dada2/issues/791) we note that the DADA2 developer argues that DADA2 should work fine with this type of data (see: https://forum.qiime2.org/t/novaseq-and-dada2-incompatibility/25865/12).

There are some other points attached as comments in the pdf of the manuscript.

Reply: Thank you for these comments. Below we reply to those not overlapping with what has already been discussed.

- Concerning the comment “Although in plain words, but that is confounding the concept of metabarcoding with that of environmental DNA” (p. 2): We agree with the reviewer and changed the wording from “that uses DNA from environmental samples” to “that can be applied to DNA from environmental samples”.

- Concerning “That's very surprising! more than 9 unseen steps in Swarm is a lot!!! I want to know what do the authors think of this.” (p. 8): 9 mismatches over 318 bp corresponds to 2.8% mismatches, which for CO1 in insects is not an unexpected high level of intra-specific diversity.

- Concerning “Does that come with the risk of dropping real clusters that match to a wrongly labelled sequence in the reference database?” (p. 9): If the wrongly labelled sequence in the reference database leads to failure at assigning the cluster to even high taxonomic ranks (with our settings, order level), it will lead to dropping the cluster. However, we believe the risk for this is rather small.

- Concerning “broken link” (p. 19): Thanks for pointing this out. It has now been fixed (https://github.com/insect-biome-atlas/paper-bioinformatic-methods)

With regards to the NEEAT algorithm, I think it poses a move in the right direction towards eliminating spurious sequences that may arise from sequencing. However, I haven't been able to explore its output, as it is embedded in HAPP and I havent managed a succesfull run of the pipeline. I think it would be useful to have a standalone version of NEEAT, so that it can be tested with other datasets and compared with other methods for spurious sequence removal.

I have installed the HAPP pipeline in two different systems: a windows laptop running WSL2 with Ubuntu 20.4 and a server running Ubuntu 20.4. In both cases, I modified the config yaml and run it with the example data provided. I would appreciate if the config/config.yaml file would be in such a way that the only thing to change would be the full path to the data. That will make it easier on the user to try the pipeline.

In my windows laptop, this was the error I got when running the pipeline:
Error in rule parse_qiime:
jobid: 3
input: results/taxonomy/vsearch/test/taxonomy.raw.tsv
output: results/taxonomy/vsearch/test/taxonomy.tsv
log: logs/parse_qiime/vsearch/test.log (check log file(s) for error details)
shell:

python /home/rgallego/.cache/snakemake/snakemake/source-cache/runtime-cache/tmp9imfwq69/file/home/rgallego/local_review/happ/workflow/rules/../scripts/parse_qiime.py results/taxonomy/vsearch/test/taxonomy.raw.tsv results/taxonomy/vsearch/test/taxonomy.tsv -r Kingdom Phylum Class Order Family Genus Species BOLD_bin > logs/parse_qiime/vsearch/test.log 2>&1

(one of the commands exited with non-zero exit code; note that snakemake uses bash strict mode!)

With full log file not adding much information.

In my server, I got the following error:
Error in rule filter_seqs:
jobid: 2
input: data/test/asv_counts.tsv,
results/chimera/test/filtered/chimera1/samplewise.uchime_denovo/nonchimeras.fasta,

/home/meg/rgallego/happ/data/test/asv_taxonomy.tsv
output: results/common/test/chimera1/samplewise.uchime_denovo/Family/taxa
log: logs/filter_seqs/test/chimera1/samplewise.uchime_denovo/Family.filter.log (check log file(s) for error details)

I understand that the troubleshooting of the pipeline is not the main focus of the manuscript, but it is hard to finish a review without a chance of seeing the output of the pipeline.

So I would mark this as major revision, not because there are substantial flaws in the manuscript, but because I would like to be able to explore the output of the pipeline. I would suggest to the authors to provide a standalone version of NEEAT, so that it can be tested with other datasets and compared with other methods for spurious sequence removal.

Reply: We're sorry to hear that the test run of HAPP failed. With the information given it is difficult to troubleshoot the problem. However, we have now done additional testing on several platforms (including WSL) and ironed out a few issues that may have been causing the errors. We kindly direct all further problems to the HAPP issue tracker on GitHub. The latest release of HAPP (v3.1.1) now also includes a standalone version of NEEAT, implemented via a separate rulesfile, which allows users to run NEEAT directly on their input data. This is also documented in the updated README.
